# The Impact of Nanoplastics on the Quality of Fish Sperm: A Review

**DOI:** 10.3390/ani16010094

**Published:** 2025-12-29

**Authors:** Hayam Djafar, Saira Naz, Maria Montserrat Rivera Del Alamo, Juan Carlos Balasch, Mariana Teles

**Affiliations:** 1 Department of Cell Biology, Physiology and Immunology, Universitat Autònoma de Barcelona, 08193 Bellaterra, Spain; hayam.djafar@autonoma.cat (H.D.); saira.naz@uab.cat (S.N.); joancarles.balasch@uab.cat (J.C.B.); 2 Department of Animal Medicine and Surgery, Faculty of Veterinary Medicine, Universitat Autònoma de Barcelona, 08193 Bellaterra, Spain; mariamontserrat.rivera@uab.cat

**Keywords:** nanoplastics, fish sperm, reproduction, motility, viability, fertilization success, aquatic toxicology

## Abstract

Pollution in aquatic ecosystems is intensifying under the combined pressures of climate change and anthropogenic contaminants, with nanoplastics (NPs) emerging as a critical threat to fish reproduction. Although extensive research has demonstrated the physiological impacts of NPs, their direct effects on sperm quality and functionality remain poorly characterized. This review synthesizes evidence from original research articles that specifically examined NPs’ impacts on fish sperm quality and related reproductive endpoints. The findings reveal that NPs consistently impair sperm motility, viability, and fertilization capacity, while inducing oxidative stress, DNA damage, mitochondrial dysfunction, and endocrine disruption. Particle size, surface chemistry, and exposure route were identified as key determinants of toxicity, with direct sperm exposure causing immediate impairments and chronic or maternal transfer exposures leading to systemic and transgenerational effects. Notably, several studies reported reduced offspring survival, altered development, and disrupted gene expression, highlighting the intergenerational risks of NPs contamination. Despite these advances, significant knowledge gaps remain, including limited research on marine wild and cultured fish species, the effects of diverse life histories on NPs toxicity, environmentally relevant exposure levels, and the combined effects of NPs with other stressors. Overall, this review underscores that fish sperm are highly sensitive to NPs pollution, with consequences that extend across generations and threaten population stability, calling for urgent mechanistic and ecologically realistic investigations.

## Simple Summary

Nanoplastics (NPs) are increasingly present in aquatic environments and are small enough to interact directly with biological cells. Fish sperm are particularly vulnerable because they are released directly into the water during fertilization and have limited protective and repair mechanisms. This review summarizes current evidence on how nanoplastics affect fish sperm quality, including motility, viability, fertilization capacity, and underlying cellular mechanisms such as oxidative stress and mitochondrial dysfunction. It also discusses indirect evidence from gonadal, hormonal, and molecular studies, while highlighting the limitations of extrapolating these effects to sperm function. Emerging findings on multigenerational effects and interactions with other environmental stressors are reviewed, emphasizing areas where data remain scarce. Finally, the review outlines key knowledge gaps and discusses the relevance of nanoplastic-induced sperm impairment for fish populations and aquaculture, where sperm quality is critical for hatchery success. Overall, this work highlights the need for more targeted, environmentally relevant studies focusing on sperm-specific endpoints.

## 1. Introduction

In an ever-changing world of increased plastic contamination, pathogen load, rising temperatures, ocean acidification, and variable marine and atmospheric circulation patterns, the resilience of freshwater and marine species depends critically on the proper activation of the endocrine hypothalamic–pituitary–gonadal axis (HPG). Among the plethora of emergent contaminants described in the last decade, plastic contamination in particular is becoming increasingly severe under changing climatic conditions [1,2]. Extreme weather accelerates the degradation of plastic fragments [3] into microplastics (MPs: ranging from 1 μm to 5 mm) and, ultimately, nanoplastics (NPs: less than 1 μm). Unlike MPs, NPs due to their reduced size can cross cellular barriers and nest in gonadal tissues, interfering with the endocrine, metabolic, and stress-related hormonal axis, and, through the production of reactive oxygen species (ROS) that induces mitochondrial and DNA damage, impairing gametogenesis, fertility rates, inducing embryo mortality or malformations, and altering overall growth [4,5,6]. Environmental stressors such as elevated temperatures or high density that may increase the NPs load, may also exacerbate the activation of the endocrine stress axis and impair gonadogenesis and spermatogenesis in fish, biasing the reproductive phenotype towards masculinization [7]. It should be noted that the upper size limit used to define nanoplastics varies across studies, with some authors considering particles <100 nm and others extending the definition up to 1000 nm, which complicates direct comparisons between studies [8,9].

NPs also interact with co-contaminants, such as metals, polycyclic aromatic hydrocarbons, pharmaceuticals, pesticides, antibiotics, plastic additives, and endocrine disruptors that can further degrade the reproductive physiology, and even the behavioral courting in fish [10], inducing oxidative stress, impairing foraging activity, immunity, and lipid metabolism, and modulating steroidogenesis and thyroid function [11,12]. NPs can also be maternally transferred to embryos as described on zebrafish (*Danio rerio*) and the ovoviviparous species *Poecilia reticulata* [13], but very few studies have addressed the intergenerational effects of NPs exposure in non-model fish species. Moreover, a recent systematic review highlighted that the effects of plastic pollutants in fish have been studied mainly in adults and females, and, to a lesser degree, in males [14].

In male fish the main physiological variables analyzed in stressful, plastic-ridden environments are spermatogenesis, sperm motility, malformation, and fertility rates, and the histopathology of the testis [15]. Spermatogenesis is a complex process dependent of the species-specific cyclic changes of the HPG axis, and the sequential activation of sex-determining genes [16], or the effect of temperature and pH in species whose sex determination depend on environmental variables acting through epigenetic modification of genes controlling gonadal differentiation and maturation [17]. However, despite increasing concern regarding NPs toxicity, a substantial gap persists in our understanding of its direct impact on fish sperm quality and functionality—a gap this review seeks to address through a critical evaluation of the existing literature.

## 2. Materials and Methods

A comprehensive literature search was conducted to identify peer-reviewed journal articles examining the toxic effects of NPs on fish sperm. Particular emphasis was placed on outcomes related to sperm motility, viability, proteomics, and transcriptomics. Search was performed in three major electronic databases: Google Scholar, Web of Science, and PubMed, and covered studies published up to August 2025 (Table 1).

The following search terms were applied, both individually and in combination:

“NPs effect on fish sperm viability”

“NPs effect on fish sperm motility”

“NPs effect on fish sperm fertilization capacity”

“NPs effect on fish reproduction”

“NPs effect on fish sperm metabolism”

For each database, the number of results generated by these keywords is summarized in Table 1.

Following the initial search, a filtering process was applied to refine the literature search, such as duplicate records identified across Google Scholar, Web of Science, and PubMed, which were removed manually. We included only original research articles with experimental data on NPs and fish reproduction (Table 2). Titles and abstracts were first screened to exclude non-fish species, review articles, opinion papers, and studies not addressing reproductive or sperm-related endpoints. Full-text screening was then applied to retain only original experimental studies that specifically assessed sperm quality parameters (e.g., motility, viability, fertilization capacity) or closely related reproductive outcomes directly linked to sperm function. Examples include studies focusing on gonadal development, hormone regulation, bioaccumulation, maternal transfer, immune responses, and co-exposure with other toxicants. After excluding such studies, only those articles specifically targeting sperm quality indicators, reproductive performance, or closely related endpoints were retained (Table 2). No formal scoring system was applied; however, studies were evaluated for experimental clarity, relevance to NPs, and the presence of clearly defined exposure conditions and endpoints. The final dataset was narrowed to nine articles because only these met all inclusion criteria, reflecting the current scarcity of studies directly addressing NPs’ effects on fish sperm. The overall literature searches and selection process followed an approach conceptually aligned with PRISMA guidelines, although implemented in a narrative rather than systematic review framework.

Studies from both marine and freshwater systems were considered. Despite the fact that these environments differ substantially in their physicochemical properties, such as salinity, ionic composition, and organic matter content, which in turn can influence NPs aggregation, stability, and bioavailability [32,33], integrating evidence from both systems allowed for a broader and more comprehensive overview of the available data. Further several studies include both microplastics and NPs but, this review focuses specifically on NPs, and results from mixed-size studies are discussed only when NPs-specific effects can be distinguished or when size-dependent differences are explicitly reported. The findings from these studies are synthesized below according to key sperm-related endpoints and biological processes, providing a structured overview of current knowledge and highlighting where gaps remain. While this review focuses on sperm quality and functionality, it is important to distinguish between studies that directly assess sperm-specific endpoints (e.g., motility, viability, fertilization capacity, mitochondrial activity, or DNA integrity) and those that evaluate broader reproductive or gonadal-level responses, such as testicular histopathology, endocrine disruption, or whole-gonad transcriptomics. The latter provide valuable mechanistic and physiological context but represent indirect proxies of sperm function. Consequently, extrapolations from gonadal alterations to sperm quality should be interpreted with caution, particularly when direct sperm measurements are not available.

## 3. Effects of NPs on the Quality of Sperm in Fish

Fish sperm, released into the surrounding water during external fertilization, are immediately exposed to contaminants, including MPs and NPs [34]. Due to small size, limited cytoplasmic content, and high membrane fluidity, sperm are particularly fragile and highly sensitive to environmental stressors [35,36]. The reviewed studies encompass both single-stressor exposures to NPs and multi-stressor scenarios in which NPs co-occur with other chemical contaminants. These two experimental approaches provide complementary insights, allowing NPs to be evaluated either as primary toxicants or as vectors and modulators of co-contaminant toxicity. Distinguishing between these roles is essential for interpreting reproductive outcomes and for understanding the ecological relevance of NPs’ exposure in complex environmental settings. In single-stressor studies, NPs alone consistently impaired sperm-related and reproductive endpoints, including spermatogenesis, sperm motility, fertilization success, and offspring development, supporting their role as primary reproductive toxicants [18,20,21]. In contrast, multi-stressor studies demonstrated that NPs can exacerbate or modify the toxicity of co-occurring contaminants, such as microcystin-LR, triclosan, or sulfamethazine, often amplifying oxidative stress, endocrine disruption, and germ cell damage [23,28,31]. These findings indicate that NPs may function not only as toxicants per se, but also as vectors or stress amplifiers in chemically complex environments.

**Figure 1:**
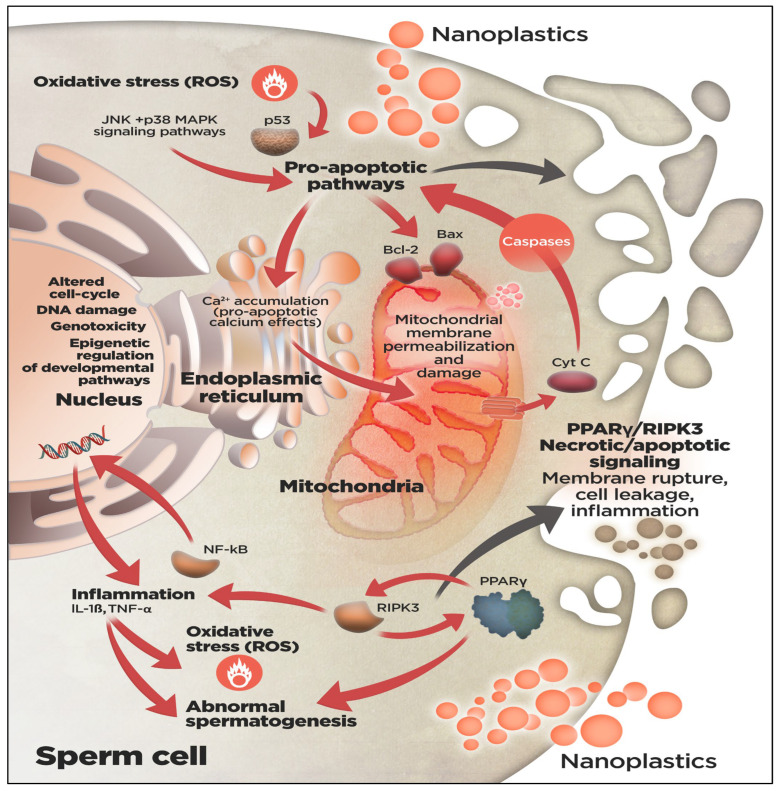
**Putative mechanisms of cellular stress due to nanoplastics (NPs) exposure in fish sperm cells**. NPs may cross the blood–testis barrier, triggering oxidative stress, inflammation, and the activation of intracellular necrotic and apoptotic pathways, ultimately impairing spermatogenesis. In fish, research on the cytotoxic effects of NPs in sperm cells remains limited and is generally restricted to a few laboratory model species (see Table 1). Furthermore, although canonical stress-related signaling pathways are broadly conserved across vertebrates, key components and their effectiveness may vary among fish species. From the studies in fish and mammalian model species, the minimum common set of molecular alterations in fish sperm cells in response to internalized NPs, as illustrated in the figure, includes: (**i**) the generation of reactive oxygen species (ROS) and pro-inflammatory mediators; (**ii**) the activation of PPARγ/RIPK3 signaling pathways involved in necrotic and apoptotic signaling; and (**iii**) the participation of p53, and JNK/p38 MAPK signaling in pro-apoptotic pathways. Upon NPs exposure, nuclear factor-κB (NF-κB) enhances the synthesis of pro-inflammatory cytokines (such as interleukin 1, IL-1β; and tumor necrosis factor alpha, TNFα) which may induce oxidative stress, DNA damage (with concomitant p53 activation), and malformations in mature sperm cells. ROS production further upregulates p53 expression, which in turn activates genes encoding cell cycle arrest factors and mediates Bax/Bcl-2-dependent permeabilization of the mitochondrial outer membrane, leading to mitochondrial damage and cytochrome c (Cyt C) release to initiate caspase-dependent apoptotic signaling. Additionally, p53 activation facilitates Ca^2+^ accumulation in the endoplasmic reticulum, augmenting the pro-apoptotic calcium effects in the mitochondria. JNK/p38 MAPK signaling pathways, primarily activated by environmental stressors and oxidative stress, regulate cellular responses to inflammatory factors, and may also induce the apoptosis machinery by modulating the expression of other apoptotic regulators, including p53, Bcl-2, and caspases. NPs exposure usually alters the metabolism of lipids and steroidogenic hormones mediated by the activation of the peroxisome proliferator-activated receptor (PPAR) signaling pathway, potentially inducing dysgenesis during sperm-cell development. The reciprocal regulation between receptor-interacting protein kinase 3 (RIPK3) and PPARγ influences the onset of programmed necrotic or apoptotic signaling cascades, contributing to inflammatory processes. Combined with endocrine disruptors and other contaminants, the exposure to NPs may also alter the epigenome of germ cells, impairing the developmental pathways.

NPs, with their reactive surfaces and ability to cross biological barriers, can adhere to or penetrate sperm cells, inducing oxidative stress [37], particularly those regulated by the nuclear factor erythroid 2–related factor 2 (Nrf2), which mediates antioxidant responses to elevated reactive oxygen species (ROS). Studies show that NPs induce oxidative stress in gonadal tissues, leading to decreased antioxidant enzyme activities and impaired spermatogenesis (Figure 1), as observed in medaka fish exposed to polystyrene NPs [30]. Excessive or prolonged oxidative stress can overwhelm Nrf2 defenses, causing mitochondrial dysfunction, disrupted energy metabolism, DNA damage, and reduced sperm motility and viability [38,39]. Proteomic and transcriptomic analyses reveal that NPs exposure dysregulates pathways related to protein transport, RNA splicing, and mTOR signaling, further impairing sperm function [40]. The limited antioxidant capacity of sperm makes them particularly vulnerable to oxidative damage, which can compromise fertilization and early embryo development [39,41]. Overall, these findings highlight oxidative stress and Nrf2 pathway dysregulation as central mechanisms of NPs-induced reproductive toxicity in fish sperm, emphasizing the need for further research on protective strategies [42].

Such impairments drastically lower fertilization success, and prolonged or chronic exposure may ultimately lead to widespread reproductive failure, threatening the survival and stability of fish populations [43]. The underlying mechanisms by which NPs impair sperm function were explored in several of the included studies, revealing a combination of oxidative stress, hormonal imbalance, and transcriptional reprogramming. In zebrafish, transcriptomic analysis following NPs exposure identified the differential expression of thousands of genes involved in meiosis, DNA repair, and chromatin remodeling [18]. These large-scale molecular changes suggest that NPs interfere with the genetic and epigenetic regulation of spermatogenesis, potentially leading to defective sperm formation. Similarly, Zheng et al. [25] reported alterations in lipid metabolism pathways and abnormal folliculogenesis in zebrafish exposed to size-dependent NPs, linking metabolic disruption to impaired gamete production. Endocrine disruption was another consistent observation.

Sun et al. [20] showed that chronic exposure to NPs altered circulating levels of estradiol, testosterone, and vitellogenin in zebrafish, indicating hypothalamic–pituitary–gonadal (HPG) axis disruption. Zhang et al. [22] confirmed these findings, demonstrating that NPs transferred maternally to offspring caused hormone imbalances and disrupted sex differentiation. At the cellular level, oxidative stress and apoptosis were recurring mechanisms. For instance, Contino et al. [24] documented increased ROS production and mitochondrial dysfunction in mussel sperm, while Sun et al. [20] observed oxidative damage in zebrafish gonads. These molecular and cellular disruptions collectively explain the observed reductions in motility, viability, and reproductive success.

Comparing across species highlights both shared vulnerabilities and species-specific differences in responses to NPs exposure. Freshwater fish models, particularly zebrafish, dominate the literature and consistently show systemic reproductive impairments, including reduced sperm motility, altered gonadal histology, endocrine disruption, and multigenerational effects [18,20,22]. In contrast, European whitefish demonstrated subtler immediate effects on fertilization capacity but significant impacts on offspring development [21]. Marine invertebrates such as mussels and oysters also exhibited acute spermiotoxicity upon direct exposure to functionalized NPs, with effects evident within hours [24,26]. These results highlight the importance of the exposure route: direct sperm exposure tends to reveal immediate impairments in motility and fertilization, whereas chronic waterborne or maternal transfer exposures reveal systemic and generational consequences. The consistent finding across taxa is that NPs impair reproductive success, but the severity and manifestation of effects differ depending on species, NPs properties, and experimental context.

### 3.1. Sperm Motility

Sperm motility was one of the most consistently assessed parameters across the included studies and appears to be a sensitive indicator of NPs toxicity. In European whitefish (*Coregonus lavaretus*), direct exposure of sperm to carboxyl-coated PS-NPs (50 nm) caused clear motility impairments in a concentration-dependent manner [21]. At higher particle concentrations, the proportion of motile sperm decreased significantly, and swimming trajectories were disrupted, suggesting that NPs’ interference with sperm surface interactions or flagellar function directly compromises motility. Interestingly, although fertilization rates were not drastically reduced, the offspring derived from exposed sperm exhibited reduced body mass and impaired swimming ability, indicating that sub-lethal effects on sperm may carry consequences into early life stages.

Similar motility impairments were observed in marine invertebrate species, such as mussels and oysters, when sperm were exposed directly to NPs. In *Mytilus galloprovincialis*, amino-modified 50 nm PS particles significantly reduced sperm motility, increased ROS production, and lowered fertilization rates [24]. Likewise, in *Crassostrea gigas*, exposure to amine- and carboxyl-functionalized PS-NPs impaired sperm motility and embryogenesis, highlighting that particle surface chemistry is a critical determinant of toxicity [26]. Similarly, in zebrafish both acute [18] and chronic [20] exposures reported motility reductions associated with altered spermatogenesis and gonadal histology. Collectively, these studies provide strong evidence that motility reduction is a primary and early endpoint of NPs toxicity in aquatic organisms, although the severity of impairment varies by species, particle size, functionalization, and exposure conditions. However, the underlying mechanisms of toxicity, the effects of dose–response relationships in normal and abnormal physiology of spermatogenesis, and the potential long-term or intergenerational consequences for fish reproduction and population dynamics remain unassessed.

### 3.2. Effects on Sperm Viability and Fertilization Capacity

Several studies have focused on the effect of NPs on sperm viability and the ability of gametes to achieve successful fertilization in aquatic species. Yaripour et al. [21] demonstrated that while fertilization rates were not significantly impaired, the resulting offspring showed reduced body mass and lower swimming performance in European whitefish (*Coregonus lavaretus*). This indicates that even when sperm retain fertilization capacity, NPs may cause subtle cellular or molecular alterations that carry forward into offspring development. In zebrafish, both multigenerational [22] and acute exposure studies such as Pujol et al. [18] reported reduced egg production, delayed hatching, and decreased larval survival, all of which can be linked to compromised sperm or oocyte quality. These results contrast with the effect of NPs in invertebrates such as mussels and oysters, where NPs exposure led to marked decreases in fertilization rates. For example, *M. galloprovincialis* sperm exposed to increasing concentrations of amino-modified NPs showed not only decreased motility but also reduced viability and mitochondrial function, leading to compromised fertilization success [24]. Similarly, *C. gigas* sperm exposed to functionalized NPs exhibited dose-dependent decreases in fertilization and abnormal embryogenesis [26]. Taken together, these findings suggest that NPs affect not only the immediate functionality of sperm but also the broader reproductive outcome, including embryo quality and early survival. However, results differ by species and exposure method, indicating a need for standardized testing across taxa.

Rising water temperatures linked to global climate change can modify the toxicity of NPs in aquatic organisms [44]. Although direct experimental evidence remains limited, rising water temperatures associated with global climate change may act as an important modifying factor of NPs toxicity in fish reproduction. However, it has been documented that elevated temperatures can increase metabolic rate, membrane fluidity, and mitochondrial activity in ectothermic organisms, potentially intensifying reactive oxygen species production and reducing cellular stress tolerance [45,46,47]. In sperm cells, which rely on tightly regulated energy metabolism and possess limited antioxidant defenses, thermal stress may therefore exacerbate NPs-induced oxidative damage and functional impairment [48]. These considerations suggest that temperature could influence the magnitude of NPS effects on sperm quality, even if it has not yet been systematically evaluated.

### 3.3. Transgenerational and Developmental Effects

Evidence for transgenerational effects of NPs in fish is currently limited to a small number of studies and is primarily restricted to parental (F0) exposure with measurable effects in the first filial generation (F1). While these studies provide clear evidence of multigenerational responses, NPs-induced reproductive toxicity has been demonstrated to extend beyond the directly exposed generation, raising hypotheses regarding possible population-level impacts. Zhang et al. [22] reported maternal transfer of NPs in zebrafish, with F1 offspring displaying disrupted gene expression in sex differentiation pathways, altered hormone levels, and impaired gonadal development. This study demonstrated that NPs can accumulate in parental tissues and be transferred to gametes and embryos, resulting in persistent developmental and reproductive alterations in offspring. Similarly, Pujol et al. [18] showed that NPs-exposed zebrafish parents produced larvae with delayed hatching, reduced survival, and abnormal cardiac function. Zheng et al. [25] also observed impaired gametogenesis and disrupted lipid metabolism that persisted in the next generation, further supporting the occurrence of multigenerational effects. Collectively, these findings indicate that NPs exposure is not limited to acute, individual-level toxicity; however, extrapolation to long-term population-level consequences remains speculative and requires further multi-generational and ecologically relevant studies.

## 4. Knowledge Gaps

**Figure 2:**
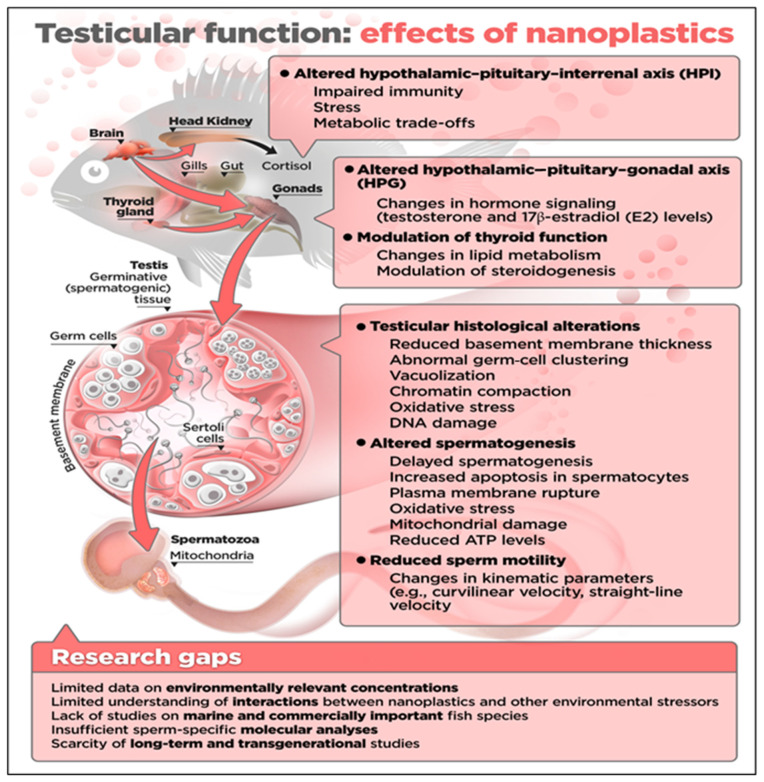
**Summary of the effects of nanoplastics in male fish**. Nanoplastics (NPs) dissolved in aquatic environments or ingested through dietary intake primarily enter fish via the gills, skin, and gastrointestinal tract, accumulating in tissues and organs. Within the testes, the small size of NPs allows them not only to be present in the seminal fluid and the testicular structures, but also to cross the cell membranes of germ cells, thereby affecting spermatogenesis. Although the effects of NPs on testicular function are species-specific, and dependent on the species analyzed, as well as on NPs size, concentration, and adherence to other compounds such as heavy metals or xenobiotics, several disruptions of reproductive homeostasis in male fish have been reported, as summarized in the figure.

Despite growing evidence, substantial gaps remain in our understanding of NPs’ effects on fish-sperm quality and reproduction (Figure 2). As in other studies of ecotoxicology in fish, there is a clear bias toward freshwater model species such as zebrafish, with marine fish species largely underrepresented despite their ecological and commercial importance. Few studies have specifically examined the effect of plastic pollution on spermatogenesis through the lens of proteomic and transcriptomic analysis in fish, and most investigations focus on whole gonadal tissue, which makes it difficult to isolate sperm-specific pathways of toxicity. Notably, the majority of studies did not directly expose sperm to NPs under conditions simulating the aquatic environment, even though sperm in natural waters are highly sensitive and fragile. Direct exposure of sperm to NPs may be critical because gametes in aquatic environments are often the first point of contact for pollutants and may respond differently than intact gonadal tissue or systemic exposure.

A major limitation across the available literature is the frequent use of high or acute NPs exposure concentrations, often in the mg/L range, which may exceed currently reported environmental levels. Field measurements generally indicate that NPs occur at much lower concentrations, typically in the ng/L to μg/L range in surface waters [49,50], although substantial uncertainty remains due to analytical limitations and the lack of standardized detection methods [51,52]. Consequently, many experimental exposure scenarios likely represent worst-case conditions rather than average environmental exposure. Another limitation of the current literature is the reliance on gonadal, hormonal, or transcriptomic endpoints in the absence of direct sperm-specific measurements, limiting conclusions on functional sperm quality and fertilization potential. Further, relatively few studies directly compare single- and multi-stressor exposure scenarios using standardized sperm-specific endpoints, limiting the ability to disentangle whether NPs act predominantly as primary toxicants or as modulators of co-contaminant effects under environmentally realistic conditions. Further from the discussion, it can be said that temperature may modulate NPs toxicity, and empirical studies explicitly examining interactions between NPs and thermal stress on sperm quality are currently lacking. Future research should prioritize multi-stressor experiments incorporating environmentally relevant temperature scenarios to better reflect climate-driven changes in aquatic systems.

Fish reproductive sensitivity to NPs is influenced by diverse life-history traits, but this area remains underexplored. Species with external fertilization and broadcast spawning release sperm directly into water, exposing sperm immediately to NPs during activation and motility, increasing vulnerability to NPs toxicity [15,53]. In contrast, species with internal fertilization, nest-guarding, or benthic spawning may have reduced or altered exposure routes and durations, potentially lowering direct sperm–NP interactions [54]. Additionally, interspecific differences in sperm longevity from seconds to minutes or longer affect the window of susceptibility to NPs, especially during the critical fertilization period [19,53]. NPs can accumulate in gonadal tissues, disrupt the hypothalamic–pituitary–gonadal axis, induce oxidative stress, and impair spermatogenesis and oogenesis, leading to reduced fecundity and abnormal offspring across fish species [15,25,53]. These findings highlight the need for targeted research on species-specific reproductive vulnerabilities to NPs, considering reproductive strategies, sperm traits, and spawning environments to better assess ecological risks and inform aquaculture management [53]. Finally, standardized protocols for assessing sperm endpoints, including motility, viability, fertilization success, and molecular markers are needed to enable cross-study comparisons and meta-analyses.

## 5. Conclusions

The evidence synthesized from the selected studies demonstrates that NPs pose a significant threat to fish reproductive health, with sperm quality emerging as a particularly sensitive target. Across multiple species, NPs exposure consistently impaired sperm motility, viability, and fertilization capacity, while also inducing molecular and cellular alterations, including oxidative stress, apoptosis, and endocrine disruption. These effects were observed not only in freshwater fish species but also in marine invertebrates such as mussels and oysters, highlighting the broad ecological relevance of NPs toxicity. Importantly, several studies revealed transgenerational consequences, showing that NPs exposure in parental generations can disrupt offspring development, hormone regulation, and gametogenesis. This underscores the long-term population-level implications of NPs pollution in aquatic environments.

The findings summarized in this review also have important implications for aquaculture and hatchery-based reproduction. In cultured species, sperm quality is a key determinant of fertilization success, larval performance, and stock sustainability. Evidence that NPs impair sperm motility, viability, and fertilization capacity suggests that male broodstock exposed to NPs either through water, feed, or contaminated rearing systems may experience reduced reproductive efficiency. Moreover, studies involving direct sperm exposure indicate that NPs present in activation water or hatchery environments could directly compromise assisted reproduction procedures, including in vitro fertilization and sperm handling protocols. These observations highlight the need for monitoring and mitigating NPs contamination in aquaculture systems, as well as for incorporating male reproductive endpoints into broodstock health assessments and risk evaluation frameworks.

Despite these findings, critical knowledge gaps remain. Most notably, very few studies directly exposed sperm to NPs under conditions reflecting the aquatic environment, even though sperm are highly fragile and may respond differently than whole gonadal tissue or systemic exposures. Additionally, there is limited information on marine fish species, environmentally relevant NPs concentrations, and the combined effects of multiple stressors. Therefore, it is recommended that further studies are conducted to investigate direct sperm exposure, mechanistic pathways, and multi-generational effects in ecologically realistic scenarios. Addressing these gaps is essential for accurately assessing the risks of NPs to fish populations and for developing strategies to mitigate their impact on aquatic ecosystems.

## Figures and Tables

**Table 1:** Number of research articles retrieved from PubMed, Google Scholar, and Web of Science using specific search keywords related to the effects of nanoplastics (NPs) on fish sperm and reproduction.

Keywords	PubMed	Google Scholar	Web of Science
NPs effect on fish sperm viability	3	742	4
NPs effect on fish sperm motility	3	832	9
NPs effect on fish sperm fertilization capacity	0	337	0
NPs effect on fish reproduction	12	3840	90
NPs effect on fish sperm metabolism	0	674	0

**Table 2:** Initial selection of 14 research articles retrieved from PubMed, Google Scholar, and Web of Science after the first-level filter (species restricted to fish and exclusion of review/opinion articles). Articles that met the final inclusion criteria (*n* = 9) are highlighted in grey.

Species	Plastic Polymer	Exposure Route	Dose	Effects (Major Findings)	Reference
**Zebrafish (*Danio rerio*)** (adult males and females).	Polystyrene nanoplastics (PS-NPs), ~45–50 nm diameter	Waterborne exposure (added directly into aquarium water)	An amount of 5 mg/L (5000 µg/L) PS-NPs. Exposure duration: 96 h (acute short-term exposure)	**Males**: Testicular damage, increased spermatocyte apoptosis, nanoplastic uptake by germ cells, strong gene dysregulation affecting meiosis and DNA repair, and reduced sperm motility. **Females**: Delayed oocyte development with minor, non-significant reduction in egg production. **Reproductive capacity**: Impaired fertilization success, shown by delayed hatching, reduced larval survival, and lower heart rate in offspring.	[18]
Marine medaka (*Oryzias melastigma*)	PS-NPs ~ 100 nm square fragments	Dietary (fish fed with feed mixed with SMZ, PS, or both)	A total of 3.45 mg/g dry feed	**SMZ**: ~35% GSI reduction, inhibited Aund self-renewal, and disrupted hormonal and apoptotic regulation. **PS**: No GSI or germ cell changes, but altered spermatogenesis gene expression. **SMZ and PS**: PS partially rescued GSI by promoting spermatogonia differentiation, without restoring Aund self-renewal	[19]
Zebrafish (*Danio rerio*)	PS-NPs, 50 nm	Waterborne exposure	An amount of 1 mg/L (21 days)	**Gonads**: PS-NPs impaired gametogenesis—fewer mature sperm and oocytes, delayed spermatogenesis and oocyte maturation. **Hormones:** Sex-hormone imbalance in both sexes, indicating endocrine disruption. **Genes**: Dysregulated BPG–axis and steroidogenesis genes. Outcome: Long-term PS-NP exposure caused oxidative stress and reproductive dysfunction	[20]
European whitefish (*Coregonus lavaretus*)	PS (specifically, 50 nm spherical PS-NPs coated with carboxyl groups, red fluorescent, Ex: 552 nm, Em: 580 nm)	Sperm from males was directly exposed to NPs in activation water for 10 s before being used to fertilize eggs	Low dose: 100 particles per spermatozoa High dose: 10,000 particles per spermatozoa	**Sperm**: High dose reduced motile sperm (more static cells) and altered swimming pattern; low dose had no effect. **Embryos**: No change in mortality; high dose accelerated hatching. **Offspring**: High dose reduced body mass and swimming performance, with no effect on length.	[21]
Zebrafish (*Danio rerio*)	PS (specifically, 100 nm spherical PS-NPs with green fluorescence, Ex: 488 nm, Em: 518 nm)	Chronic waterborne exposure of female adults (F0) for 42 days; offspring (F1) raised in clean water but exposed via maternal transfer (PS-NPs detected in F1 embryos and larvae)	Low dose: 200 μg/L High dose: 2000 μg/L	**Bioaccumulation**: Dose-dependent buildup with maternal transfer to embryos and larvae. **F0 reproduction**: High dose reduced egg production and ovary maturity, with E2 ↓, T ↑, and disrupted HPG–axis genes. **F1 effects:** Faster hatching, higher larval heart rate, altered neuroendocrine and sex-differentiation gene expression, but no change in adult sex ratio or ovary structure.	[22]
Male zebrafish (*Danio rerio*)	PS; microplastics [PSMPs] of 5 μm diameter; NPs [PSNPs] of 80 nm diameter)	Waterborne (chronic exposure in aerated glass tanks with renewal of exposure solutions every 3 days); also in vitro exposure on mouse GC-2 spermatocytes	PSMPs/PSNPs: 1 mg/L Microcystin-LR (MCLR): 0, 5, 25 μg/L Combinations: MCLR (5 or 25 μg/L) and PSMPs (1 mg/L) or PSNPs (1 mg/L) (Exposure duration: 45 days for in vivo; various for in vitro)	**Synergy**: PS (especially PSNPs) increased MCLR bioavailability, causing stronger combined testicular toxicity than single exposures. **Mechanisms**: Co-exposure amplified oxidative stress, DNA damage, mitochondrial dysfunction, and cell-cycle arrest in spermatogenic cells. **Reproduction**: Reduced GSI, severe testicular damage, and high risk of impaired spermatogenesis and fertility.	[23]
Mediterranean mussel (*Mytilus galloprovincialis*)	PS (specifically, 50 nm amino-modified PS-NPs [nPS-NH_2_]; 100 nm NPs also tested but showed no significant effects)	Waterborne exposure of sperm in controlled aquatic microenvironments (in vitro; sperm incubated with NPs for 30 min prior to fertilization)	Low: 0.1 mg/L Medium: 1 mg/L High: 10 mg/L (100 nm NPs tested at same concentrations but showed no significant effects)	**Size-dependent toxicity**: 50 nm nPS-NH_2_ (not 100 nm) induced ROS, mitochondrial damage, and ATP loss in sperm. **Sperm quality:** High doses reduced motility and caused membrane and DNA damage. **Reproduction**: Fertilization success markedly decreased at ≥1 mg/L.	[24]
Zebrafish (*Danio rerio*)	PS-NPs with diameters of 80 nm, 200 nm, and 500 nm)	Waterborne exposure (chronic exposure in aquariums with water renewal every 2 days)	Single concentration: 0.5 mg/L (Exposures tested for each size: 80 nm, 200 nm, 500 nm; duration: 21 days for F0 adults, with F1 embryo assessment)	**F0 reproduction:** Delayed spermatogenesis, abnormal follicle growth, reduced GSI, strongest effects with 500 nm NPs. **F1 offspring**: Impaired development—higher mortality, delayed hatching (worst with 500 nm). **Mechanisms:** Disrupted PPAR signaling and lipid metabolism in ovaries, resembling PCOS. Size effect: 500 nm NPs caused greatest bioaccumulation and reproductive toxicity.	[25]
Pacific oyster (*Crassostrea gigas*)	Polystyrene (specifically, 50 nm polystyrene beads with amine [50-NH_2_] or carboxyl [50-COOH] functional groups)	Waterborne exposure (in vitro; oyster spermatozoa incubated with NPs in filtered seawater for 1 h)	Range: 0.1 to 25 mg/mL Key tested concentrations: 0.1, 1, 10, and 25 mg/mL (for both 50-NH_2_ and 50-COOH beads)	**Sperm motility**: 50-NH_2_ (10 mg/mL) and 50-COOH (25 mg/mL) strongly reduced motile sperm and velocity; lower doses had no effect. **Reproduction**: 50-NH_2_ decreased embryogenesis by 59%; 50-COOH had no significant effect. **Mechanism**: 50-NH_2_ caused membrane-mediated spermiotoxicity; 50-COOH effects were due to aggregation/physical blockage.	[26]
Oriental river prawn (*Macrobrachium nipponense*)	Polystyrene (PS; specifically, polystyrene microplastics [PS-MPs] with a size of 5 μm)	Waterborne (acute exposure for larvae; chronic exposure for adults via immersion in contaminated water)	Acute exposure (larvae): 0, 2, 20 mg/L (24-h exposure) Chronic exposure (adults): 0, 2, 20 mg/L (4-week exposure)	**Larvae (acute):** Reduced survival and heart rate; no direct reproductive effects. **Adult males (chronic):** Impaired testicular development, oxidative stress, apoptosis, and disrupted steroid hormones. **Reproduction**: Lower hatching success, higher F1 malformations. **F1 offspring:** Reduced survival and immune function; bioaccumulation of PS-MPs observed.	[27]
Zebrafish (*Danio rerio*)	Polystyrene (PS; microplastics [MPS] of 5.8 μm diameter; NPs [NPS] of 46 nm diameter)	Waterborne exposure (chronic exposure via immersion in contaminated water)	MPS and NPS: 0, 20 μg/L Triphenyl phosphate (TPhP): 0, 200 μg/L Combinations: TPhP (200 μg/L) + MPS (20 μg/L) or NPS (20 μg/L) (Duration: 21 days)	**Acute toxicity:** MPS/NPS did not alter TPhP LC50. **Reproduction:** TPhP enlarged liver/gonads; NPS worsened effects, MPS minimal impact. **Histology:** Co-exposure impaired spermatogenesis and oogenesis, more severe with NPS. **Hormones:** NPS altered E2/T and Vtg levels in a sex-dependent manner. **Outcome:** TPhP and PS reduced egg production, fertilization, and hatchability; NPS amplified reproductive toxicity most.	[28]
Zebrafish (*Danio rerio*)	Polystyrene (specifically, polystyrene microplastics with a size of 5 μm)	Waterborne exposure (continuous immersion in contaminated water)	Low: 10 mg/L Medium: 100 mg/L High: 1000 mg/L (Duration: 21 days)	**Oxidative stress**: ROS increased in gonads and liver at ≥100 mg/L; no effect at 10 mg/L. **Apoptosis**: Elevated in testes at 1000 mg/L via p53 pathway; lower doses unaffected. **Histology**: Testicular basement membrane thinning at 1000 mg/L; no changes at lower doses.	[29]
Japanese medaka (*Oryzias latipes*)	Polystyrene (specifically, polystyrene NPs [NPs] with a diameter of 100 nm)	Waterborne exposure (chronic exposure via immersion in contaminated water)	Low: 10 particles/L Medium: 10^4^ particles/L (10,000 particles/L) High: 10^6^ particles/L (1,000,000 particles/L) (Duration: 3 months)	**Immune & oxidative stress:** Reduced LZM, SOD, CAT, GPx, and increased MDA in gonads at ≥10^4^ particles/L. **Gonads**: High dose (10^6^ particles/L) inhibited spermatogenesis and oogenesis; lower doses had no effect. **Survival**: Three-month exposure decreased survival, worst at 10^6^ particles/L.	[30]
Zebrafish (*Danio rerio*)	Polystyrene (specifically, polystyrene NPs [NPs] with a diameter of 50 nm)	Waterborne exposure (chronic exposure via immersion in contaminated water)	Polystyrene NPs (NPs): 0, 10 μg/L Triclosan (TCS): 0, 0.3 μg/L Combinations: TCS (0.3 μg/L) + NPs (10 μg/L) (Duration: 21 days, with F1 generation assessment)	**GSI**: TCS reduced GSI; NPs alone had no effect; co-exposure further decreased GSI, especially in males. **Histology**: TCS impaired spermatogenesis and oogenesis; NPs worsened testicular and ovarian damage. **Hormones**: TCS disrupted E2/T balance; NPs amplified male E2 increase and female T elevation. **Genes**: Co-exposure dysregulated sex hormone and vitellogenin genes in both sexes. **Reproduction (F1):** Lower fertilization, hatching, and larval survival, strongest from male exposure.	[31]

## Data Availability

Data will be made available on request.
